# Maternal oral contraceptive pill use and the risk of atopic diseases in the offspring: A systematic review and meta-analysis

**DOI:** 10.1097/MD.0000000000019607

**Published:** 2020-04-17

**Authors:** Xue-Feng Bai, Zheng-Xiang Wu, Chun-Hong Zhao, Yong Wu, Chang-Shun Fei, Li-Qin Zhang, Zhao-Hui Chen

**Affiliations:** a Otolaryngology Head Neck Surgery, Tongxiang First People's Hospital; b Department of Pediatrics, Tongxiang Second People's Hospital, Tongxiang; c Department of Otolaryngology Head and Neck Surgery, Affiliated Hospital of Hangzhou Normal University, Hangzhou, Zhejiang, China.

**Keywords:** allergy, atopy, contraception, hormone, pregnancy, wheeze

## Abstract

Studies of maternal oral contraceptive pill (OCP) exposure and the offspring's risk of atopic diseases are of current interest due to concerns about widespread use of OCP before or during pregnancy.

We evaluated whether maternal OCP exposure is associated with an increased risk of atopic diseases by reviewing the literature and performing a meta-analysis. The PubMed and Embase databases were searched to identify potential studies for inclusion. Three common atopic outcomes were included: asthma, eczema, and rhinitis.

We found 693 titles, abstracts, and citations, and 6 studies were included in this analysis. A meta-analysis revealed that maternal OCP exposure was associated with higher odds of asthma (odds ratio [OR] 1.1; 95% confidence interval [CI] 1.02–1.19; *P* = .014), rhinitis (OR 1.34; 95% CI 1.07–1.68; *P* = .011) during childhood, whereas there was no association with eczema (OR 1.17; 95% CI 0.81–1.68; *P* = .383). This analysis was limited by the small number of studies included and the limited adjustments for the possible confounders in the studies.

Current evidence suggests that maternal OCP exposure increases the risk for respiratory allergic diseases (asthma and rhinitis) in the offspring, but not for eczema. Given the few studies included, future larger, prospective studies that control for important confounders are needed to verify our findings.

## Abstract 2

Supplemental Digital Content is available in the text

## 1 Introduction

Atopic diseases are the most common chronic diseases in children, and their incidence has increased over the past decade.^[1,2]^ Atopic diseases are highly heritable; however, accumulating evidence suggests that environmental factors are involved in their pathogenesis.^[3]^ In utero and perinatal exposure may predispose offspring to these diseases^[4,5]^; thus, recognition of the maternal risk factors for atopic diseases and implementation of appropriate strategies may facilitate their prevention.

At the beginning of this epidemic about 40 to 50 years ago, oral contraceptive pills (OCP) were frequently used by fertile-aged women.^[6]^ However, most pregnancies occur shortly after OCP discontinuation or during OCP use, which could influence hormone levels during pregnancy.^[7]^ Therefore, maternal OCP use may be a risk factor for atopic diseases, and atopic disease-associated levels of progesterone and estrogen during pregnancy are known.^[8]^ However, it remains controversial whether maternal OCP use is associated with an increased risk for atopic diseases. A number of observational studies^[9–14]^ have investigated this matter. One large study^[13]^ reported no increased risk for asthma in children of mothers exposed to OCP, whereas 2 smaller studies^[12,14]^ did. In addition, 2 studies^[9,11]^ found that maternal OCP use was marginally associated with an increased risk for rhinitis, while 1 Japanese study reported a 90% increase in the risk for ever developing rhinitis. Finally, only 1 study^[9]^ reported an increased risk for eczema. The inconsistent conclusions may be attributed to the heterogeneity of these studies regarding factors such as sample size, exposure time, and types of atopic disease. Thus, these factors must be analyzed separately.

Given the frequency of exposure to OCP during pregnancy, evaluation of the relationship between OCP use and the risk for atopic diseases in children is important. Therefore, we conducted a systematic literature review and meta-analysis of the association between fetal exposure to OCP and the development of atopic diseases.

## 2 Methods

The guidelines developed by the Meta-analysis of Observational Studies in Epidemiology Group were used during the preparation of this meta-analysis^[15]^ (Appendix S1). All steps in the literature search, study selection, quality assessment, data extraction, and statistical analyses were performed independently by 2 investigators from different subspecialties. Disagreements were resolved by discussion with a third author, and articles to be included were selected by consensus. No ethical approval was required for this review as all data were already published in peer-reviewed journals. No patients were involved in the design, conduct or interpretation of our review.

### 2.1 Literature search

We conducted a comprehensive literature search of the PubMed and Embase databases up to December 2019 for peer-reviewed English-language studies. We selected synonymous terms (including MeSH terms) to develop a search strategy (Table S1). The reference lists of the retrieved articles were manually searched for additional potentially eligible studies.

### 2.2 Study selection

Observational studies that met all of the following inclusion criteria were eligible for this meta-analysis: cross-sectional, case-control, or cohort study; inclusion of a group of mothers who did not take OCP as a reference group for comparison; association between maternal OCP use and the risk for atopic disease was investigated; and provision of raw data (to enable estimation of risk). To ensure accuracy, 2 authors working independently selected the studies.

### 2.3 Data extraction and quality assessment

A specifically designed Excel spreadsheet was used by 3 authors to record the basic characteristics of the included studies. Any discrepancies in the abstracted data were resolved by consensus. The following variables were collected from each study: author, year of publication/study period, country, sample demographics, number of subjects in each group, information regarding OCP exposure, diagnostic criteria for atopic diseases, and statistical adjustments. Two authors independently assessed the risk for bias using the Newcastle–Ottawa scale,^[16]^ which was developed to assess the quality of nonrandomized studies. The Newcastle–Ottawa scale scores observational studies on 3 dimensions relevant to research quality: selection and comparability of the subjects, and ascertainment of the outcome of interest; all questions have a value of 1. A score ≥7 was used to identify high-quality articles. However, only studies with a quality score ≥6 were included in the analyses. The ranks of each study are listed in Tables S2 and S3.The outcome of interest was the risk of developing 3 atopic diseases (asthma, rhinitis, and eczema) following maternal exposure to OCP. Meta-analyses were performed if the included studies provided more than 2 estimates.

### 2.4 Statistical analyses

All analyses were conducted using Stata (ver. 12.0; Stata Corp., College Station, TX). The data were pooled using a random-effects model.^[17]^ To investigate potential sources of heterogeneity, the subgroup analyses were stratified using the type of OCP, timing of OCP exposure, and whether the study adjusted for a family history of atopic diseases. For association studies, the odds ratio (OR) and 95% confidence interval (CI) were calculated for dichotomous outcome variables. All risk estimates included in the pooled analyses were from the most fully adjusted multivariable model. Heterogeneity among studies was assessed using the *χ*^2^ test and *I*^2^ statistic; an *I*^2^ value of >50% or a *P*-value of <.05 for the *Q*-statistic indicated significant heterogeneity.^[18]^ A funnel plot was not generated because fewer than 10 studies were included.^[17]^

## 3 Results

### 3.1 Search results

**Figure 1:**
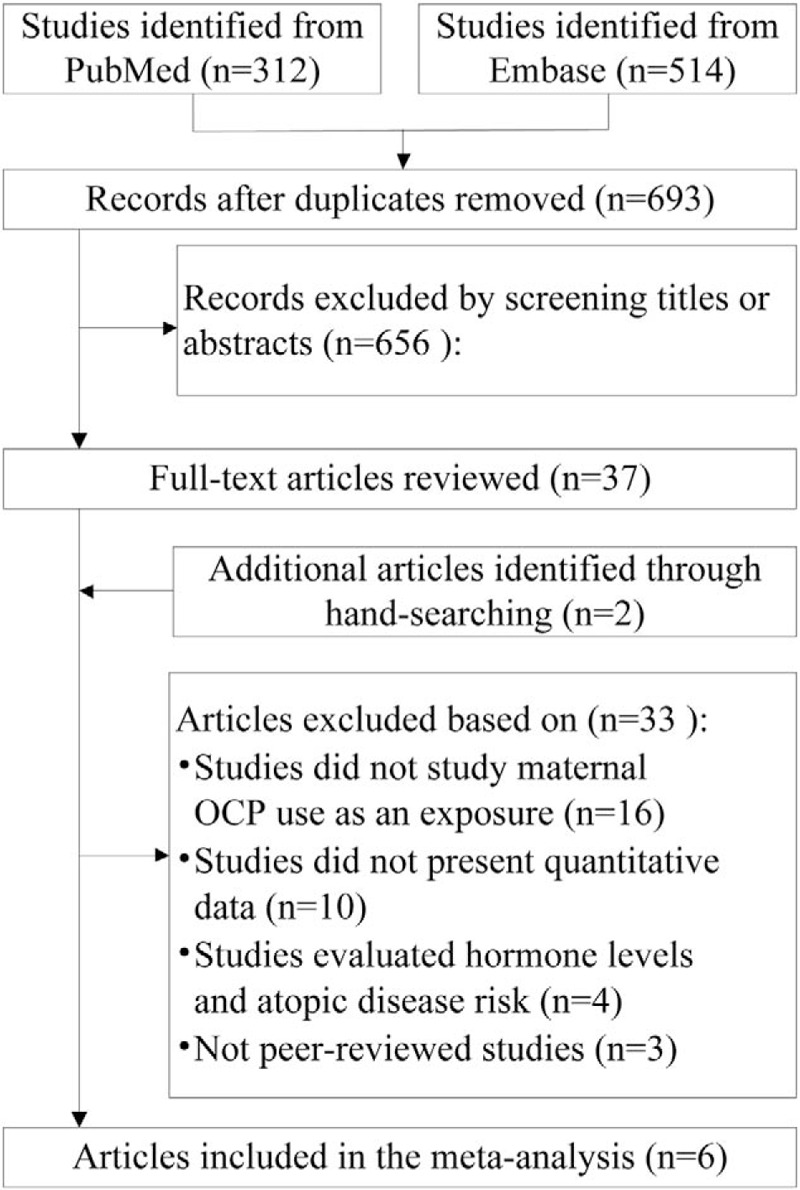
Flow chart of the studies considered and finally selected for review.

The preliminary search yielded 693 publications after excluding duplicates, of which 656 were rejected after reading the titles and abstracts. Of the remaining 39 abstracts, a subsequent full-text screening resulted in rejection of 33 studies. Finally, 6 studies that met the inclusion criteria were assessed for quality. The literature search and selection process is shown in Figure 1.

### 3.2 Characteristics of the included studies

The characteristics of the 6 studies included in this analysis are listed in Table 1. The year of publication was 2003 to 2016, and the sample size was 980 to 60,225; the pooled total was 78,871. Of the included studies, 3 were case-control studies,^[11–13]^ 2 were cross-sectional studies,^[9,10]^ and 1 was a cohort study.^[14]^ Most of the studies used a questionnaire to assess OCP use, while 1^[12]^ derived the data from birth registries and prescription registries. Four studies were conducted in Western populations (German,^[9]^ Finland,^[11]^ the United Kingdom,^[12]^ and Norway^[13]^); the other 2 studies were performed in North America^[10]^ and East Asia.^[14]^ In the methodological quality assessment, all studies scored greater than 7 and were deemed high quality. The scores are listed in Tables S2 and S3.

**Table 1:**
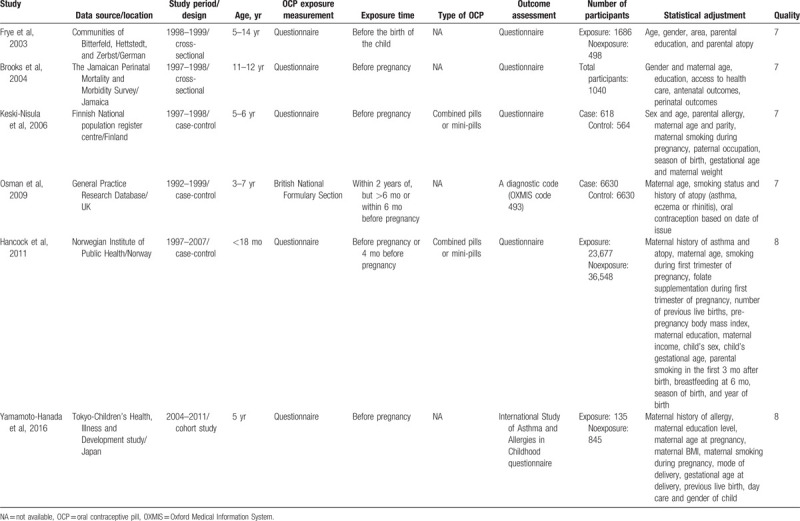
Characteristics of the included studies.

### 3.3 Maternal OCP exposure and the risk for atopic diseases

**Figure 2:**
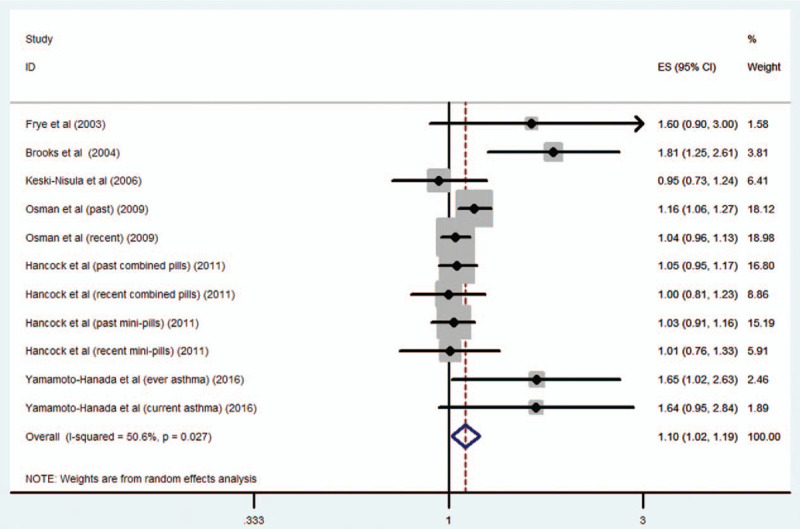
Maternal OCP exposure and risk of asthma in the offspring. OCP = oral contraceptive pill.

All analysis results are shown in Table 2. Six studies^[9–14]^ with 12 estimates including 10,025 cases reported the risk for asthma in relation to maternal OCP use; the combined OR of asthma risk was 1.1 (95% CI 1.02–1.19; *P* = .014) (Fig. 2). Sensitivity analyses were performed by removing the study with the largest effect size from the OR summary analyses. In the sensitivity analyses, the OR of the remaining studies (OR 1.07–1.21) was similar to the pooled OR of all studies. When the analysis was limited to studies providing data adjusted for a family history of atopic diseases, the pooled OR was 1.08 (95% CI 1.01–1.15; *P* = .019). When our analyses were based on exposure time, an increased risk of asthma was observed for OCP use before pregnancy (OR 1.09; 95% CI 1.01–1.18; *P* = .023), but not for OCP use within 6 months before pregnancy (OR 1.03; 95% CI 0.96–1.11; *P* = .061). When our analyses were based on the type of OCP, no increased risk of asthma was found for mini-OCP use (OR 1.01; 95% CI 0.91–1.12; *P* = .59) or combined-OCP use before pregnancy (OR 1.04; 95% CI 0.95–1.13; *P* = .32).

**Table 2:**
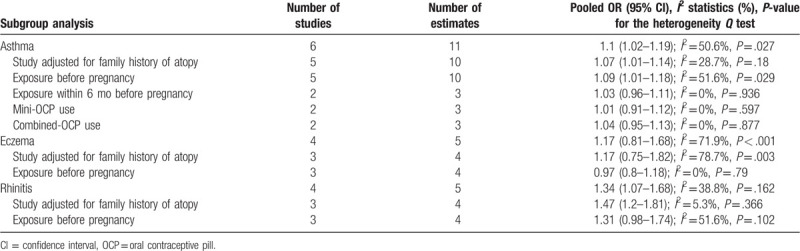
Meta-analysis for studies included in the analysis.

**Figure 3:**
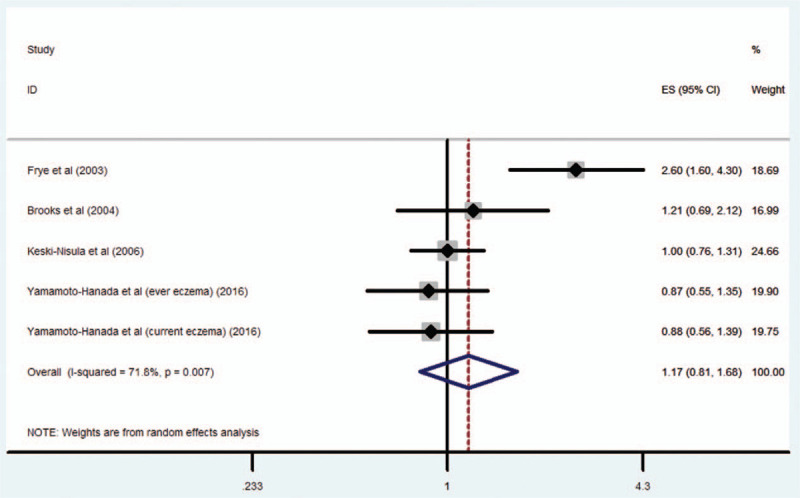
Maternal OCP exposure and risk of eczema in the offspring. OCP = oral contraceptive pill.

Four studies^[9–11,14]^ with 5 estimates including 1275 cases reported the risk for eczema in relation to maternal OCP use; the combined OR of eczema risk was 1.17 (95% CI 0.81–1.68; *P* = .393) (Fig. 3). However, there was considerable heterogeneity across studies (*I*^2^ = 71.8%). When the analysis was limited to studies providing data adjusted for a family history of atopic diseases, the pooled OR was 1.17 (95% CI 0.75–1.82; *P* *=* .491). When the analysis was limited to OCP use before pregnancy, the pooled OR was 0.97 (95% CI 0.8–1.18; *P* = .788).

**Figure 4:**
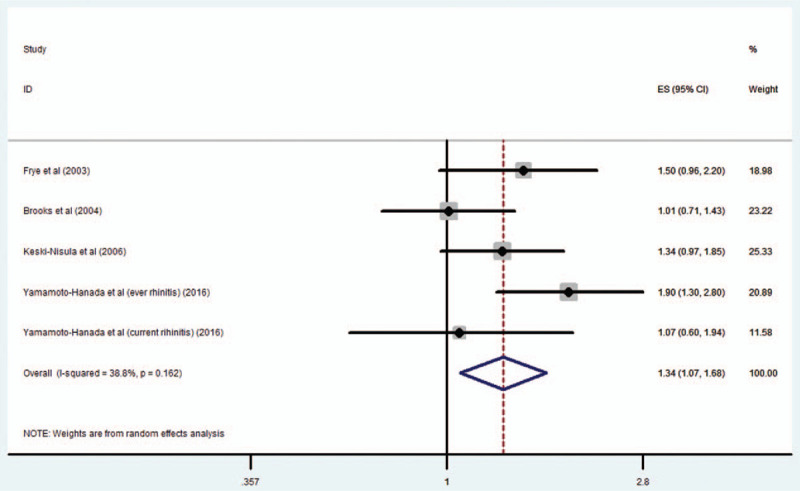
Maternal OCP exposure and risk of rhinitis in the offspring. OCP = oral contraceptive pill.

Four studies^[9–11,14]^ with 5 estimates including 970 cases reported the risk for rhinitis in relation to maternal OCP use; the combined OR of rhinitis risk was 1.34 (95% CI 1.07–1.68; *P* = .011) (Fig. 4). Moderate heterogeneity was found across studies (*I*^2^ = 38.8%). When the analysis was limited to studies providing data adjusted for a family history of atopic diseases, the pooled OR was 1.47 (95% CI 1.2–1.81; *P* < .001). When the analysis was limited to OCP use before pregnancy, the pooled OR was 1.31 (95% CI 0.98–1.74; *P* = .064).

## 4 Discussion

To our knowledge, this is the first systematic review and meta-analysis of the association between maternal OCP use and the risk for atopic diseases in the offspring. The results indicate that maternal use of OCP is associated with a moderate increase in the risk for respiratory atopic diseases (asthma and rhinitis) in the offspring, after adjusting for several confounding effects. However, maternal OCP use was not associated with eczema in the offspring.

Several hypotheses for the increased risk for atopic diseases due to maternal use of OCP have been proposed, but the underlying mechanisms remain elusive. Adjustments of the endocrine system after pregnancy are essential for fetus growth and development.^[19,20]^ Although OCP are inactivated and eliminated within 4 weeks, effects of hormones have been noted months and even years after cessation of OCP use.^[21–23]^ However, a prolonged endocrine effect seems less likely given that fetuses are naturally exposed to higher levels of progesterone than those used in OCP. Brooks et al^[10]^ hypothesized that OCP use could cause an immunologic shift from a T-helper 1 to a T-helper 2 environment in the mother, which was related to the pathogenesis of atopic diseases. Therefore, we speculated that a change in the immune response rather than the indirect endocrine effects of OCP use plays a major role in the development of atopic diseases in children. Note that maternal OCP exposure did not increase risk for eczema; this may be explained by studies reporting that OCP modulate immune responses throughout the body, particularly at mucosal sites.^[24]^ Other data suggest that sex hormones can alter the composition of the vaginal microbiota,^[25]^ which is the main source of gut microbes for infants delivered vaginally.^[26]^ The gut microbiota is involved in the regulation of immune function and the development of atopic diseases.^[27]^ Thus, the observed association between OCP and the risk for respiratory atopic diseases may be mediated by complex interactions among hormones, the maternal vaginal microbiome, and immunity.

Atopic diseases such as asthma and rhinitis have high heritability, estimated in twin studies to be 60% to 80%.^[28]^ A family history of atopic disorder is related to the development of atopic diseases in offspring; thus, investigation of the association between maternal OCP use and atopic diseases in children should consider a family history of atopic conditions. The ideal control for genetic confounding is a sibling-matched study; however, no included study adopted this method. Only 1 study^[10]^ provided an estimate without adjusting for a family history of allergy. In this review, sensitivity analyses showed that the significant association between maternal OCP use and the risk for respiratory atopic disease remained after adjustment for confounding genetic factors. Note; however, that the sample sizes were small, which limited the power of these analyses and the accuracy of the results.

Another important consideration is the somewhat arbitrary, and thus problematic, definition of the OCP use window. The risk for inflammatory bowel diseases associated with OCP use reportedly decreases after discontinuation of the drug,^[29]^ raising a similar question. Unfortunately, the time–effect relationship could not be analyzed due to the dearth of data and use of inconsistent definitions. Nonetheless, 2 studies^[12,13]^ explored the influences of exposure time on the risk for asthma; only Osman et al^[12]^ reported an increased risk among past users. One of these studies showed that long-term users were at increased risk for asthma compared with short-term users. Therefore, further investigations are required to clarify the time– and dose–effect relationships of OCP use with the risk for atopic diseases. It would also be interesting to investigate whether maternal OCP use is associated with more severe forms of atopic disease.

Although maternal OCP use is associated with a modestly increased risk for respiratory atopic disease, the population impact of OCP-associated respiratory atopic disease is likely to be substantial because of the large number of users. Our data contribute to the existing literature, suggesting that women planning to get pregnant should stop the OCP sooner so as to not increase the risk of respiratory atopic diseases in the offspring. Despite growing interest in the relationships between maternal OCP use and childhood allergic outcomes, the epidemiological evidence so far remains insufficient to provide clinical guidance. The results of this meta-analysis provide further justification for studies that consider the type or duration of maternal OCP exposure to clarify the contribution of OCP to the risk of atopic diseases in children.

The strength of this study lies in its rigorous systematic review and meta-analysis of all relevant reports to date. Furthermore, we conducted sensitivity or additional analyses to test the robustness of the results and to control for confounding. Nonetheless, our review had several major limitations. The most important is the small sample size, specifically when different analyses were used, as reflected in the small effect size. Therefore, the association between maternal OCP use and atopic disease risk may be overestimated and further investigation is needed. A second limitation was the various measurements of atopic diseases used in the included the studies. A well-designed questionnaire and standardized data acquisition were used instead of diagnostic criteria to verify cases in 5 included studies. Thus, use of different diagnostic definitions of atopic diseases may lead to substantially different values even in the same population. Further research should be based on accurate definitions of atopic diseases. Third, the observational studies are susceptible to detection bias because children who have been exposed to OCP may be more likely to undergo more extensive health assessment, possibly leading to a higher rate of atopic diseases. This bias may affect the true association. Finally, the existence of high heterogeneity could influence the robustness of our findings although we pooled the data using a random-effects model. The source of heterogeneity can be explained by differences in the baseline characteristics, exposure definition, and time and duration of exposure. Future studies should consider these confounders.

The findings of this meta-analysis suggest that maternal OCP use is associated with a moderate increase in the risk for respiratory atopic diseases, although not that of eczema, in the offspring; however, causality remains to be confirmed. In view of the challenges and difficulties in evaluating this association, further studies on this matter are needed.

## Acknowledgments

The authors thank all investigators who offered help in the systematic meta-analysis.

## Author contributions

**Conceptualization:** Zhao-Hui Chen.

**Data curation:** Zheng-Xiang Wu.

**Formal analysis:** Chun-Hong Zhao, Hui-Biao Li.

**Investigation:** Yong Wu.

**Methodology:** Chang-Shun Fei.

**Project administration:** Xue-Feng Bai, Zhao-Hui Chen.

**Software:** Li-Qin Zhang.

**Writing – original draft:** Xue-Feng Bai, Zhao-Hui Chen.

**Writing – review & editing:** Zhao-Hui Chen.

## Supplementary Material

Supplemental Digital Content

## Supplementary Material

Supplemental Digital Content

## Supplementary Material

Supplemental Digital Content

## Supplementary Material

Supplemental Digital Content
