# Supplementary material for: Maternal oral contraceptive pill use and the risk of atopic diseases in the offspring: A systematic review and meta-analysis
Source: Medicine (Baltimore). 2020 Apr 17;99(16):e19607. doi: 10.1097/MD.0000000000019607 (PMC7220114; doi:10.1097/MD.0000000000019607)
Supplement: Supplemental Digital Content [file medi-99-e19607-s001.doc]

**Appendix S1: MOOSE Checklist**

| **Criteria** | | **Brief description of how the criteria were handled in the meta-analysis** |
| --- | --- | --- |
| **Reporting of background should include** | |  |
| √ | Problem definition | While there is a growing body of evidence that suggests that prenatal oral contraceptive pills (OCPs) exposure is linked with atopic diseases in the offspring, however, this relationship was inconsistent in different studies, and it remains to be summarized quantitatively. |
| √ | Hypothesis statement | Research from animal models has gradually documented the connection between the levels of pro-gesterone and estrogen during pregnancy and atopic diseases in offspring; therefore, it is plausible to hypothesize that prenatal OPC exposure may also augment the risk of atopic diseases |
| √ | Description of study outcomes | Atopic diseases (asthma, eczema, rhinitis) in the offspring |
| √ | Type of exposure or intervention used | prenatal OPC exposure |
| √ | Type of study designs used | Cohort studies, case-control studies and cross-sectional studies. |
| √ | Study population | Children are born exposure to OPCs and controls |
| **Reporting of search strategy should include** | |  |
|  | Qualifications of searchers (eg, librarians and investigators) | - |
| √ | Search strategy, including time period included in the synthesis and keywords | PubMed and EmBase databases up to Dec 2018.  **Table S1** |
| √ | Effort to include all available studies, including contact with authors | References of all retrieved articles and recent reviews were reviewed. |
| √ | Databases and registries searched | PubMed and EmBase databases |
| √ | Search software used, name and version, including special features used (eg, explosion) | We did not employ a special search software. |
| √ | Use of hand searching (eg, reference lists of obtained articles) | References of all retrieved articles and recent reviews were reviewed. |
| √ | List of citations located and those excluded, including justification | Details of the literature search process are outlined in the flow chart. |
| √ | Method of addressing articles published in languages other than English | We placed restrictions on English. |
| √ | Method of handling abstracts and unpublished studies | The search process was restricted upon peer-reviewed articles. |
|  | Description of any contact with authors. | - |
| **Reporting of methods should include** | |  |
| √ | Description of relevance or appropriateness of studies assembled for assessing the hypothesis to be tested | The inclusion criteria are presented in the “Search strategy and Study selection” section. |
| √ | Rationale for the selection and coding of data (eg, sound clinical principles or convenience) | Study characteristics were extracted independently by two researchers (XFB and ZHC). The most adjusted estimate was included when a study reported more than one risk estimate. |
| √ | Documentation of how data were classified and coded (eg, multiple raters, blinding, and inrerrater reliability) | Data were independently extracted and analyzed by two investigators (CHZ and YW) and final decision was reached by consensus. |
| √ | Assessment of confounding (eg, comparability of cases and controls in studies where appropriate) | Table 1 presents the adjustment factors for each study. |
| √ | Assessment of study quality, including blinding of quality assessors; stratification or regression on possible predictiors of study results | The quality of each study was assessed by two investigators (CSF and LQZ), using the Newcastle-Ottawa Scale. |
| √ | Assessment of heterogeneity | The *Q*-statistic and *I*-squared (*I*^2^) statistic were used to explore the heterogeneity among studies. |
| √ | Description of statistical methods (eg, complete description of fixed or random effects models, justification of whether the chosen models account for predictors of study results, dose-response models, or cumulative meta-analysis) in sufficient detail to be replicated | Description of methods of meta-analyses, sensitivity or additional analyses, and assessment of publication bias are detailed in the “Statistical analysis” section. |
| √ | Provision of appropriate tables and graphics | One main tables and three supplemental tables are provided. One flow chart and three forest plots appear in the main text. |
| **Reporting of results should include** | |  |
| √ | Graph summarizing individual study estimates and overall estimate | Figures 2-4; FigureS1; “Results” section |
| √ | Table giving descriptive information for each study included | Table 1 |
| √ | Results of sensitivity testing (eg, subgroup analysis) | “Results” section |
| √ | Indication of statistical uncertainty of findings | “Discussion” section; |
| **Reporting of discussion should include** | |  |
| √ | Quantitative assessment of bias (eg, publication bias) | “Results” section and “Discussion” section. |
| √ | Justification for exclusion (eg, exclusion of non-English-language citations) | The details of the exclusion of studies are shown in Flow chart. |
| √ | Assessment of quality of included studies | Studies have been analyzed by the quality. |
| **Reporting of conclusions should include** | |  |
| √ | Consideration of alternative explanations for observed results | We discussed that some included studies didn't make adjustment for other factors or only make adjustment for a few important factors, thus, we cannot exclude chance, residual or unmeasured confounding as alternative explanation for our findings. |
| √ | Generalization of the conclusions (ie, appropriate for the data presented and within the domain of the literature review) | We discussed that some of included studies were based on the exposure time, type of atopic diseases or confounding factors they adjusted for maternal or parental atopic diseases. This may lead to a potential heterogeneity. |
| √ | Guidelines for future research | We discussed that more studies are needed to assess the risk of atopic diseases in children exposure to OPCs. We also discussed that more mechanistic studies are needed to further explain the association between prenatal OPC exposure and increased risk of atopic diseases in children. |
| √ | Disclosure of funding source | The authors received no specific funding for this work. |
