# Supplementary material for: Maternal oral contraceptive pill use and the risk of atopic diseases in the offspring: A systematic review and meta-analysis
Source: Medicine (Baltimore). 2020 Apr 17;99(16):e19607. doi: 10.1097/MD.0000000000019607 (PMC7220114; doi:10.1097/MD.0000000000019607)
Supplement: Supplemental Digital Content [file medi-99-e19607-s002.docx]

**Table S1: Search strategy in Pubmed and Embase**

1. pregnancy (MeSH term)
2. mothers (MeSH term)
3. pregnant (key word)
4. gestational (key word)
5. prenatal (key word)
6. perinatal (key word)
7. gestation (key word)

8. maternal

9. asthma

10. rhinitis

11. dermatitis

12. eczema

13. conjunctivitis

14. urticarial

15. immunoglobulin E

16. allergy

17. atopy

18. atopic

19. contraceptive

20. 1 OR 2 OR 3 OR 4 OR 5 OR 6 OR 7 OR 8

21. 9 OR 10 OR 11 OR 12 OR 13 OR 14 OR 15 OR 16 OR 17 OR 18

22. 19 AND 20 AND 21
