# Supplementary material for: Maternal oral contraceptive pill use and the risk of atopic diseases in the offspring: A systematic review and meta-analysis
Source: Medicine (Baltimore). 2020 Apr 17;99(16):e19607. doi: 10.1097/MD.0000000000019607 (PMC7220114; doi:10.1097/MD.0000000000019607)
Supplement: Supplemental Digital Content [file medi-99-e19607-s003.docx]

| **Table S2** NOS for Assessment of Quality of Included Studies: Cross-sectional or Case-Control Studies | | | | | | | | | |
| --- | --- | --- | --- | --- | --- | --- | --- | --- | --- |
| Study | Selection | | | | Comparability | | Exposure | | |
|  | Is the case definition adequate | Representativeness of cases | Selection of controls | Definition of controls | Study controls for age/sex | Study controls for at least 3 additional factors | Ascertainment of exposure | Same method of ascertainment of exposure | Nonresponse rate |
| Frye et al, 2003 | ★ | **★** | ★ | ★ | ★ | ★ | **―** | ★ | **―** |
| Brooks et al, 2004 | **★** | ★ | ★ | ★ | ★ | ★ | **―** | ★ | **―** |
| Keski-Nisula et al, 2006 | ★ | ★ | ★ | ★ | ★ | ★ | ― | ★ | **―** |
| Osman et al, 2009 | ★ | ★ | ★ | ★ | ★ | ★ | ★ | ★ | **―** |
| Hancock et al, 2011 | ★ | ★ | ★ | ★ | ★ | ★ | ― | ★ | **―** |
