# Supplementary material for: Maternal oral contraceptive pill use and the risk of atopic diseases in the offspring: A systematic review and meta-analysis
Source: Medicine (Baltimore). 2020 Apr 17;99(16):e19607. doi: 10.1097/MD.0000000000019607 (PMC7220114; doi:10.1097/MD.0000000000019607)
Supplement: Supplemental Digital Content [file medi-99-e19607-s004.docx]

| Table S3 NOS for Assessment of Quality of Included Studies: Cohort Studies | | | | | | | | |
| --- | --- | --- | --- | --- | --- | --- | --- | --- |
| Study | Selection | | | | Comparability | | Outcomes | |
|  | Representativeness of exposed cohort? | Selection of the nonexposed cohort? | Ascertainment of exposure? | Demonstration that outcome of interest was not represent at the start of the study | Comparability of Cohort | Assessment of outcome | Was follow-up long enough for outcomes to occur | Adequacy of follow up of cohorts |
| Yamamoto-Hanada et al, 2016 | ★ | ★ | ― | ★ | ★★ | ★ | ★ | ★ |
